# Structural Basis of Substrate Selectivity of *E. coli* Prolidase

**DOI:** 10.1371/journal.pone.0111531

**Published:** 2014-10-29

**Authors:** Jeremy Weaver, Tylan Watts, Pingwei Li, Hays S. Rye

**Affiliations:** Department of Biochemistry and Biophysics, Texas A&M University, College Station, Texas, United States of America; National Institute for Medical Research, Medical Research Council, London, United Kingdom

## Abstract

Prolidases, metalloproteases that catalyze the cleavage of Xaa-Pro dipeptides, are conserved enzymes found in prokaryotes and eukaryotes. In humans, prolidase is crucial for the recycling of collagen. To further characterize the essential elements of this enzyme, we utilized the *Escherichia coli* prolidase, PepQ, which shares striking similarity with eukaryotic prolidases. Through structural and bioinformatic insights, we have extended previous characterizations of the prolidase active site, uncovering a key component for substrate specificity. Here we report the structure of *E. coli* PepQ, solved at 2.0 Å resolution. The structure shows an antiparallel, dimeric protein, with each subunit containing N-terminal and C-terminal domains. The C-terminal domain is formed by the pita-bread fold typical for this family of metalloproteases, with two Mg(II) ions coordinated by five amino-acid ligands. Comparison of the *E. coli* PepQ structure and sequence with homologous structures and sequences from a diversity of organisms reveals distinctions between prolidases from Gram-positive eubacteria and archaea, and those from Gram-negative eubacteria, including the presence of loop regions in the *E. coli* protein that are conserved in eukaryotes. One such loop contains a completely conserved arginine near the catalytic site. This conserved arginine is predicted by docking simulations to interact with the C-terminus of the substrate dipeptide. Kinetic analysis using both a charge-neutralized substrate and a charge-reversed variant of PepQ support this conclusion, and allow for the designation of a new role for this key region of the enzyme active site.

## Introduction

Prolidases, also known as Xaa-Pro dipeptidases, are metalloproteases that catalyze the hydrolysis of dipeptides containing a C-terminal proline residue. These enzymes are conserved in prokaryotes and eukaryotes, including not only single-celled organisms, such as yeast, but also humans and higher plants [1]–[8]. In higher organisms, prolidase serves a critical role in the recycling of collagen, as the penultimate products of collagen catabolism include the dipeptides Ala-Pro and Gly-Pro [9]–[11]. In humans, specific mutant alleles of prolidase have been linked to a wide array of physiological problems, which are known collectively as prolidase deficiency [10]–[13]. Despite the importance of human prolidase and the disease states associated with various mutations of the gene, knockout and knockdown studies in several eukaryotic model organisms have yet to reveal an essential role for prolidase [14]–[17]. Therefore, further studies are required for insight into the role of prolidase in collagen metabolism and human health.

In contrast to the human enzyme, there are no observable phenotypes for *Escherichia coli* prolidase mutants [18]. While a physiological role for prolidase in bacteria remains to be established, the enzyme is known to possess protective activity against toxic organophosphates [6], [19]–[21]. The *E. coli* enzyme may also play a role similar to that of human prolidase – the breakdown of dipeptides stemming from protein catabolism – or an additional, regulatory role [22]. In support of this theory, *Mycoplasma* species possess Xaa-Pro peptidases [23]–[25]. These bacteria, which evolved to retain only those cellular functions essential to their parasitic lifestyle, import most amino acids and lipids from the host cell [26]. The fact that *Mycoplasma* retain an enzyme for cleaving Xaa-Pro bonds suggests that prolyl peptide catabolism plays a broad and generally important physiological role.

Prolidases share a number of conserved sequence and structural features. These enzymes possess an N-terminal domain and a C-terminal catalytic domain, and form dimers through contact between both domains in a head-to-tail arrangement [5]. The catalytic site features a binuclear metal cluster in the center of a pita-bread fold that is a canonical feature of this family of enzymes [22], [27]. While the identity and configuration of the coordinating ligands are conserved, the types of metals found in the active site vary widely, though manganese, cobalt and zinc appear to be the most common metals used [28]. Such metal variability has been observed in other pita-bread fold proteins [29], [30]. Interestingly, the human prolidase can utilize magnesium, though to significantly lower extent than manganese – a feature not commonly seen in other prolidases [2], [4], [28], [31]. Crystal structures of various prolidases, particularly those with bound substrates or inhibitors, have provided important structural insights into how these enzymes bind substrate peptides and metals, though few members of this enzyme family have been thoroughly examined biochemically.

Members of the pita-bread fold family of proteins, which also includes other metalloproteases, share a number of sequence-specific features that permit robust structure/function prediction, despite the varying substrate specificities of different enzymes [27]. The first prolidase structure solved was from the archaea *Pyrococcus furiosus* [5], [32], which confirmed that prolidases possess many of the structural features common to the pita-bread fold superfamily. However, four large regions of primary structure, ranging from 13–25 amino acids in length, are found in the human prolidase that do not appear in the *P. furiosus* sequence [5]. Some of these regions are also absent from related pita-bread fold members, including methionine aminopeptidases, which cleave N-terminal methionine residues, as well as proline aminopeptidases, which cleave N-terminal residues that are followed by proline, from both bacterial and human sources [27], [33].

Interestingly, the peptide regions absent in *P. furiosus* are present in prolidases from Gram negative bacteria, including *E. coli* and *Alteromonas sp.* [5], and include eleven residues highly conserved between humans and these two bacteria. *E. coli* PepQ, the only prolidase found in this organism [18], was previously characterized for activity against dipeptides, organophosphates and other small molecules [6], though the lack of an atomic structure for PepQ has prevented a detailed comparison to other prolidases. Examination of the *Alteromonas* prolidase structure, however, reveals an arginine residue reaching into the active site from one of the additional peptide segments. This residue appears to be involved in positioning a structured water molecule and other active site residues and metals, and has been postulated to interact with the C-terminus of the substrate dipeptide [20], an interaction similar to that seen in a shifted location for proline aminopeptidase [34]. Because proline aminopeptidases cleave tripeptides, the positioning of this residue may have evolved to specify substrate length in pita-bread fold proteins.

Here we report the structure of *E. coli* PepQ, showing it to have the predicted pita-bread fold. We examine its ability to utilize various active site metals, including magnesium. Furthermore, we compare its sequence and structural similarity to proline aminopeptidase and other prolidases, showing that the position of the conserved arginine has, in fact, moved throughout evolution, likely to accommodate substrate peptide length. We further characterize the role of this arginine, demonstrating that it plays a critical role in substrate dipeptide binding.

## Materials and Methods

### Cloning, Expression and Purification of PepQ and PepQ Mutants

The PepQ gene was PCR amplified from purified, chromosomal *E. coli* DNA, using primers adding a 5′-NdeI restriction site and a 3′-XhoI restriction site. The PCR product was sub-cloned into the pET21a vector (Novagen) and the sequence of this construct was verified by DNA sequencing. The R370E mutation of the PepQ gene was created via site-directed mutagenesis of the wild-type construct and was verified by DNA sequencing. Either 6 or 12 L of LB-Amp (100 mg/L) were inoculated 1∶500 with overnight cultures of BL21[DE3] cells transformed with either the wild-type or R370E PepQ plasmid. Upon reaching an A_600_ = 0.6–0.8, expression was induced with the addition of IPTG to a concentration of 400 µM. After four hours, the cells were centrifuged and the pellets were resuspended in cell disruption buffer (20 mM Tris, pH 8, 1 µM MnCl_2_, 20% (w/w) sucrose, 4 mM DTT). Cells were lysed using a gas-driven cell-disruptor (Microfluidics Corporation; Newton, MA) and clarified by ultracentrifugation. The supernatant was loaded onto a fast-flow Q (GE Healthcare) anion exchange column. The column was washed with Buffer A (50 mM Tris, pH 7.4, 1 µM MnCl_2_, 2 mM DTT) and washed with Buffer A containing 100 mM NaCl. A linear gradient was then developed from 100 mM to 500 mM NaCl. The fractions of the greatest PepQ purity were concentrated by precipitation with 70% (w/v) ammonium sulfate. The pellet was resuspended in a small volume of Buffer A containing 500 mM ammonium sulfate and loaded on a phenyl-sepharose hydrophobic interaction column (GE Healthcare). After washing with Buffer A containing 1 M ammonium sulfate, a linear gradient was developed from 1 M to 300 mM ammonium sulfate. Fractions of the greatest PepQ purity were again concentrated by precipitation with 70% (w/v) ammonium sulfate. The pellet was resuspended with a small volume of Buffer A and dialyzed against Buffer B (25 mM Tris, pH 7.4, 25 mM KCl, 1 µM MnCl_2_, 2 mM DTT). Following addition of glycerol to 15% (v/v), the sample was aliquoted, snap frozen with liquid nitrogen and stored at −80°C. Thawed samples showed no detectable loss of enzymatic activity.

### Cloning, Expression and Purification of Alanine Dehydrogenase (AlaDH)

The AlaDH gene was PCR amplified from purified, chromosomal *Bacillus subtilis str. 168 *DNA, using primers adding a 5′-NcoI restriction site (which required a mutation in the second codon, which was later reverted with site-directed mutagenesis) and a 3′-XhoI restriction site. The PCR product was sub-cloned into the pETDuet vector (Novagen) and the sequence of this construct was verified by DNA sequencing. Protein expression was conducted in 6 L of LB-Amp (100 mg/L) inoculated 1∶500 with overnight cultures of BL21[DE3] cells transformed with the AlaDH plasmid. Upon reaching an A_600_ = 0.6–0.8, expression was induced with the addition of IPTG to a concentration of 400 µM. After four hours, the cells were centrifuged and the pellets were resuspended in cell disruption buffer (20 mM Tris, pH 8, 0.5 mM EDTA, 20% (w/w) sucrose, 4 mM DTT). Cells were lysed, clarified and loaded onto a fast-flow ion exchange column, as described above. The column was washed with Buffer C (50 mM Tris, pH 7.4, 0.5 mM EDTA, 2 mM DTT) containing 150 mM NaCl. A linear gradient was then developed from 150 mM to 500 mM NaCl. Fractions of the greatest AlaDH purity were concentrated by precipitation with 70% (w/v) ammonium sulfate. The pellet was then resuspended in a small volume of Buffer C containing 1 M ammonium sulfate. The sample was then loaded on a phenyl-sepharose hydrophobic interaction column (GE Healthcare). After washing with Buffer C containing 900 mM ammonium sulfate, a linear gradient was developed from 900 to 650 mM ammonium sulfate. Fractions of the greatest AlaDH purity were concentrated by precipitation with 70% (w/v) ammonium sulfate. The pellet was resuspended with a small volume of Buffer C and dialyzed against Buffer D (25 mM Tris, pH 7.4, 25 mM KCl, 0.5 mM EDTA, 2 mM DTT). Following addition of glycerol to 15% (v/v), the sample was aliquoted, snap frozen with liquid nitrogen and stored at −80°C. Thawed samples showed no detectable loss of enzymatic activity.

### Crystallization and Refinement of PepQ

The PepQ sample was buffer-exchanged into 50 mM Tris, pH 7.4, 5 mM MgCl_2_ and 5 mM DTT at a final concentration of 12 mg/ml. The protein was crystallized by the hanging drop vapor diffusion method at 4°C using 20% PEG MME 5000 in 0.1 M Bis-Tris buffer at pH 6.5. The crystals were transferred stepwise to a cryobuffer containing 30% PEG 400, 20% PEG MME 5000, 0.1 M Bis-Tris at pH 6.5 and flash frozen in liquid nitrogen. The diffraction data were collected at beamline 7.1 at the Stanford Synchrotron Radiation Lightsource (SSRL) using a Quantum 315R CCD detector. The diffraction data were processed with the HKL2000 package [35]. The structure was determined by molecular replacement using Phaser in the Phenix package [36]. A homology model of PepQ generated using Swiss-Model based on the crystal structure of *Alteromonas macleodii* OpaA structure (PDB 3RVA) [20] was used as search model. The model was fine-tuned with Coot [37] and refined using the Phenix package [36]. Statistics of data collection and refinement are shown in Table 1.

**Table 1:** Statistics of crystallographic analysis for pepQ.

PDB Entry	4QR8
**Data collection**	
Space group	P2_1_2_1_2_1_
*Cell dimensions*	
*a*, *b*, *c* (Å)	72.57, 97.44, 126.94
*α*, *β*, *γ* (°)	90.0, 90.0, 90.0
Resolution (Å)	2.00 (2.03 to 2.00)^1^^,^ 2
3 *R*_sym_ or *R*_merge_	11.6% (0.695)
*I/*σ*I*	18.0 (2.0)
Completeness (%)	97.0 (92.3)
Redundancy	4.5 (3.5)
**Refinement**	
Resolution (Å)	2.0
No. reflections	59597
4 *R*_work_/5 *R*_free_	17.39%/21.1%
*No. atoms*	
Protein	7052
Ligand/ion	4
Water	1314
*B-factors*	
Protein	20.3
Ligand/ion	19.6
Water	31.2
*R.m.s. deviations*	
Bond lengths (Å)	0.004
Bond angles (°)	0.80

1 One crystal was used to collect each of the dataset.

2 Values in parentheses are for highest-resolution shell.

3 R_sym_ = ∑_h_∑_i_|*I*_i,hkl_–<*I*_hkl_>|/∑_hkl_∑_i_ |*I*_i,hkl_|, where *I*_hkl,i_ is the intensity measured for a given reflection with Miller indices h, k, and l, and <*I*_hkl_> is the mean intensity of that reflection.

4 R_work_ = ∑||F_o_|−|F_c_||/∑|F_o_|, where F_o_ and F_c_ are the observed and calculated structure-factor amplitudes, respectively.

5 R_free_ was calculated as R_work_ using a randomly selected subset (10%) of unique reflections not used for structure refinement.

### Metal Usage

Metal usage of PepQ was directly monitored by the decrease in absorbance at 222 nm upon cleavage of the substrate peptide bond [6]. *E. coli* PepQ was diluted to 12.5 µM into 50 mM Tris, pH 7.4 and 10 mM EDTA. Following incubation at 25°C for 30 min, this solution was then diluted 25-fold into 25 mM Tris, pH 7.4 containing either a divalent metal (1 mM), EDTA (5 mM) or no additional component. Samples were incubated at 25°C for an additional 10 min. This sample was then diluted 10-fold with 10 mM Tris, pH 8 and the substrate dipeptide AlaPro (TCI-America). The reaction was immediately assayed at 25°C. The final concentration of PepQ was 50 nM and AlaPro was 0.25 mM in a final volume of 1 mL. All assays were conducted using a Perkin Elmer Lambda 35 spectrophotometer with a PCB 1500 water Peltier temperature control system.

### Docking Simulations

Preparation of structure files and docking was done as described [38]. In brief, substrate and protein structure files were prepared using MGL Tools, in which polar hydrogens were added and flexible bonds were designated. Autodock Vina was then used to simulate the interaction of the small molecules with the active site of PepQ.

### Enzyme Quaternary Structure

The stability of the dimeric structure of wild-type and R370E PepQ was determined using analytical gel filtration. PepQ (10 nM) in 50 mM Tris, pH 7.4, 50 mM KOAc, 10 mM Mg(OAc)_2_ and 2 mM DTT was injected on a Superose 6 gel filtration column (GE), equilibrated in the same buffer, with a constant flow rate of 0.4 mL/min driven by an HPLC unit with a binary pump (Waters). The tryptophan fluorescence (excitation at 280 nm, emission at 340 nm) of the sample was measured using an in-line, post-column fluorescence detector (Waters).

### Enzyme Stability

The thermodynamic stability of wild-type and R370E PepQ was determined by the red-shift in the tryptophan fluorescence peak as the protein unfolds with increasing concentrations of the chemical denaturant guanidinium-HCl. PepQ (50 nM) was incubated at room temperature for 60 minutes in solutions of 50 mM Tris, pH 7.4, 10 mM Mg(OAc)_2_, 2 mM DTT and varying concentrations of guanidinium-HCl. The tryptophan fluorescence was measured using a PTI fluorometer with excitation at 295 nm and emission from 315–375 nm. Solutions of buffer and guanidinium at each concentation, without protein, were also measured to account for changes in scattered light. The peak maximum and corresponding wavelength was determined using Microsoft Excel (MAX and VLOOKUP functions).

### Enzyme Kinetics

The PepQ reaction rate was monitored by coupling the hydrolysis of the dipeptide AlaPro to the NAD-dependent oxidation of alanine [39]. These reactions were conducted in a 1 mL volume in 50 mM Tris, pH 8 and 20 mM Mg(OAc)_2_ at 25°C with varying concentrations of AlaPro-COOH (TCI America) or AlaPro-CONH_2_ (Chem-Impex), supplemented with 1 µM AlaDH and 2 mM NAD^+^ (Chem-Impex). The increase in absorbance at 340 nm was monitored as NADH was produced. All assays were conducted using a Perkin Elmer Lambda 35 spectrophotometer with a PCB 1500 water Peltier temperature control system.

## Results

### *E. coli* Prolidase Possesses an Expanded Sequence

**Figure 1:**
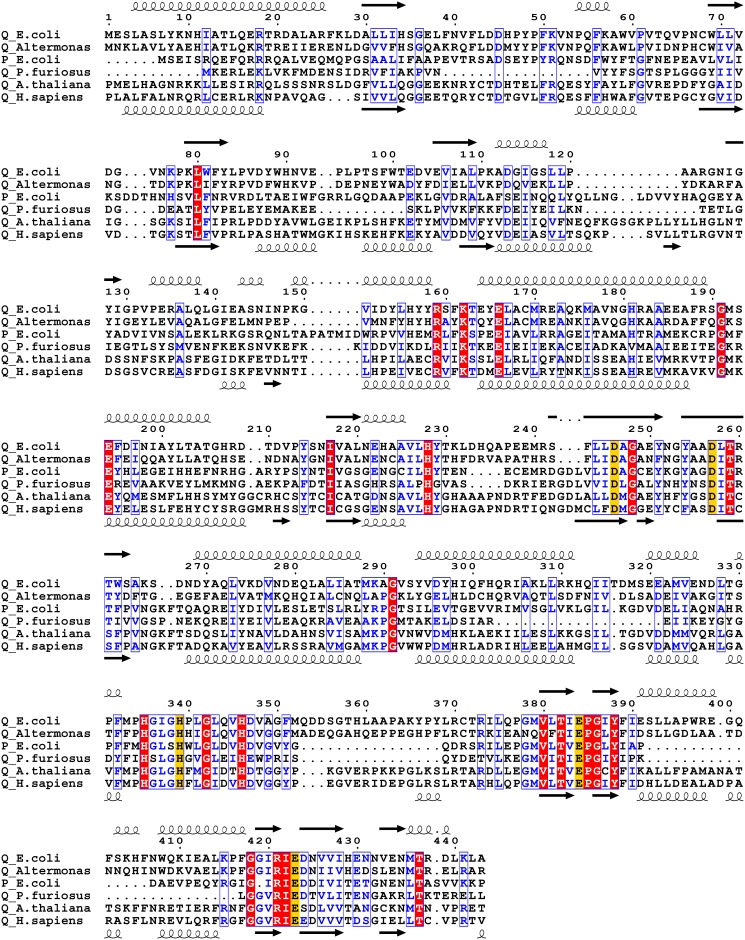
Sequence alignment of prolidases. Sequence alignment of *E. coli* PepQ (accession number P21165) with eukaryotic and prokaryotic pita-bread fold enzymes was performed using CLUSTALW [60] and graphically organized with ESPript [61]. Completely conserved residues are highlighted in red and highly conserved residues or regions are boxed and shown in blue. Metal-chelating residues are highlighted with yellow. Numbering shown is for *E. coli* PepQ. Secondary structure assignments shown above the alignment are those from *E. coli* PepQ, while those shown below the alignment are from human PepD. The aligned proteins (with percent identity/similarity to *E. coli* PepQ, along with the number of aligned positions shown in parentheses; followed by the accession number of the sequence) are: *Alteromonas sp.* PepQ (50/67, 441), Q44238; *E. coli* PepP (31/46, 330), P15034; *Pyrococcus furiosus* PepQ (24/40, 337), P81535; *Arabidopsis thaliana* Xaa-Pro Dipeptidsae (34/51, 292), Q8L780; *Homo sapiens* PepD (29/45, 466), P12955. The degree of identity and similarity was determined by two-sequence alignment with BLAST [62].

To examine the extent of sequence conservation in the *E. coli* prolidase, PepQ, we collected primary structure information from organisms with sequenced genomes, including both higher plants and animals. Upon alignment (Figure 1), many regions of *E. coli* PepQ show sequence similarity (boxed) and identity (shaded) with the sequences of human and plant prolidase, illustrating the conservation of various elements of this protein family. Overall, *E. coli* PepQ shows high sequence identity (∼30%) and similarity (∼50%) with the eukaryotic prolidases. Furthermore, the *E. coli* sequence shows good coverage of the human gene, with only one region of 10–15 residues missing (Figure 1, between *E. coli* residues 120–125). Although these additional regions may be shifted in our alignment, in a previous alignment [5], four regions of at least ten residues appeared in *E. coli* and human prolidase, but did not appear in *P. furiosus* prolidase (*E. coli* residues 35–53, 303–321, 360–372 and 391–415). In these regions, eleven residues (*E. coli* residues Gly36, Asp45, Phe50, Leu309, Ser319, Glu321, Leu369, Arg370, Glu391, Leu393 and Leu394) are conserved. Of these residues, all but two (Ser319 and Glu391) are also conserved among *E. coli*, humans and *Arabidopsis* (Figure 1). While shorter than ten residues, another additional region appears in all of the sequences, but not in *P. furiosus* PepQ – an N-terminal loop extension (94–101), though this region does not include any conserved residues.

**Figure 2:**
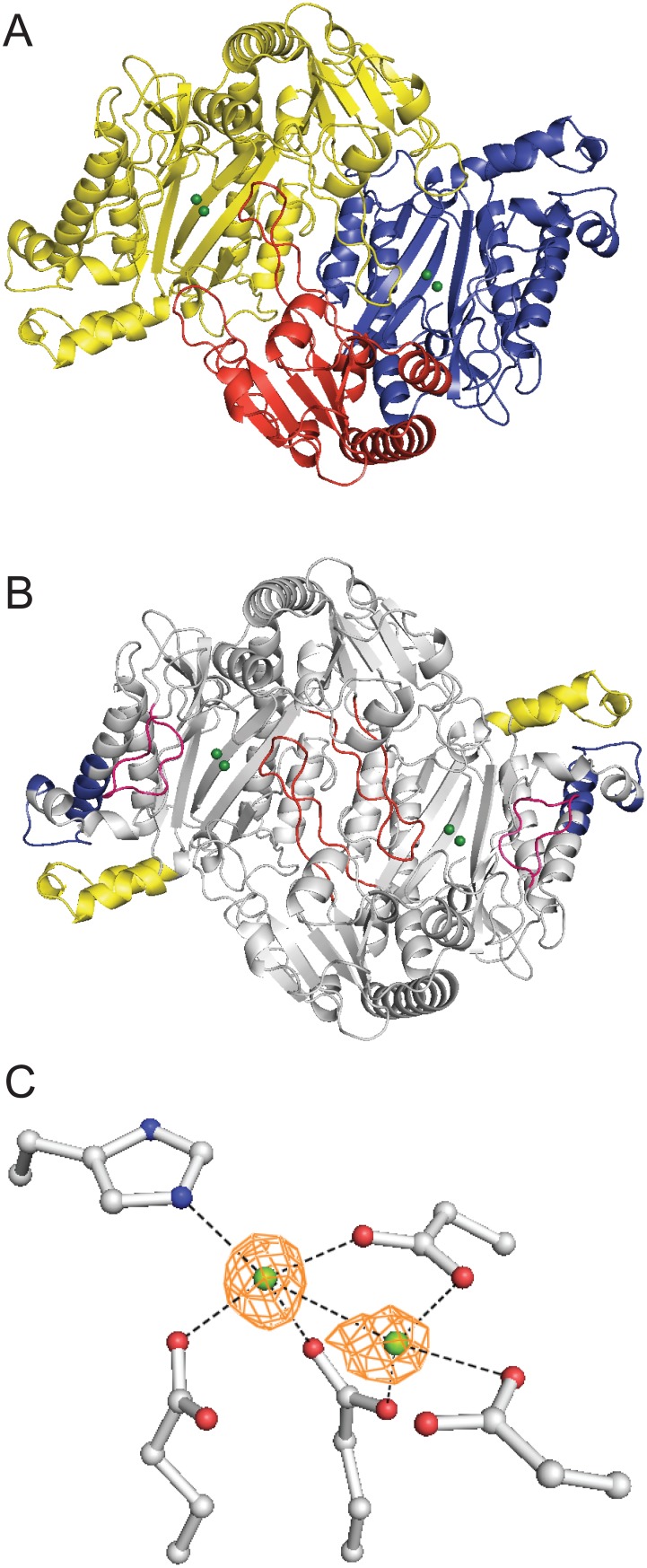
PepQ forms a canonical pita-bread fold with binuclear active site. (A) The PepQ dimer (PDB entry 4QR8) is shown with one monomer shown in yellow and one monomer colored by domain: N-terminal (residues 1–159, red) and catalytic (160–443, blue). The magnesium ions are colored green. The image was rendered in PyMOL [63]. (B) The PepQ dimer with new regions of sequence (those not in *P. furiosus*) highlighted (residues 35–53, red; 303–321, blue; 360–372, pink; 391–415, yellow). (C) Electron density shows conserved active site residues coordinating two magnesium ions.

To better understand the potential significance of sequence conservation between the *E. coli* and human prolidases, we solved the structure of the bacterial enzyme at 2.0 Å resolution (Figure 2A, Table 1). The protein is comprised of two sections – an N-terminal domain and a C-terminal catalytic domain. The catalytic domain features the predicted, canonical pita-bread fold common to this family of enzymes. At the center of the pita-bread fold is the active site, containing two metal ions chelated by five residues (metals shown in green). The asymmetric unit contains a single PepQ dimer, which is the native oligomeric structure of this protein [6], arranged head-to-tail with inter-dimer contacts made between both domains. With tertiary and quaternary features appearing as expected, we next focused our analysis on the regions of sequence not found in *P. furiosus* and the residues in those regions that are conserved in other sequences.

The conserved regions in PepQ consist of two helicies and two loop structures, and three of these structural features are in the catalytic domain (Figure 2B). The N-terminal loop (highlighted in red) makes significant contact with the same loop from the other subunit. The loop in the catalytic domain (highlighted in pink) extends into the active site. The two helices in the catalytic domain (highlighted in blue and yellow) are on the outside edge of the domain, with both helices in contact with each other and one also in contact with the loop in the catalytic domain (pink). Given the location of these regions of sequence, it is not surprising that only two of the nine residues found in regions absent from *P. furiosus* (but conserved from *E. coli* into the eukaryia), are located near the active site of the enzyme (Asp45 and Arg370). The Arg370 equivalent residue in *Alteromonas* (also Arg370) has been predicted to play a role in organizing water in the active site and, possibly, interacting with the C-terminus of the substrate dipeptide [20]. Asp45, which reaches into the active site of one monomer from a loop region in the N-terminal domain of the other monomer, is seen in *E. coli* to be within interaction distance of Arg370, with the charged ends of the side chains approximately 3.5Å apart. The conservation of this interaction suggests co-evolution of these residues in support of additional known interactions in the active site.

The active site of *E. coli* PepQ also features canonical metal binding residues, Asp246, Asp257, His339, Glu384 and Glu423, chelating two metal ions. Because PepQ was crystallized in buffer containing magnesium, the density found in this region is most likely derived from magnesium ions (Figure 2C). Additionally, the mF_0_-DFc difference map shows greater density for one of the two metal ions (chelated by His339), consistent with reports from other pita-bread fold peptidases that this binding site has a higher affinity for metal ions [34], [40]. The decreased occupancy at the second metal site is surprising, given that the magnesium concentration during crystallization was in the millimolar range. This observation suggests that the affinity for magnesium of either PepQ in general, or this site in particular, is not as high as seen for the preferred manganese ion in related proteins, reported to be in the low- or sub-micromolar range [22], [34], [41]. However, metal binding by prolidase does not necessarily convey enzymatic activity, leaving the functionality of magnesium-bound PepQ unresolved.

### *E. coli* PepQ Can Utilize Mutliple Metals for Catalysis

**Figure 3:**
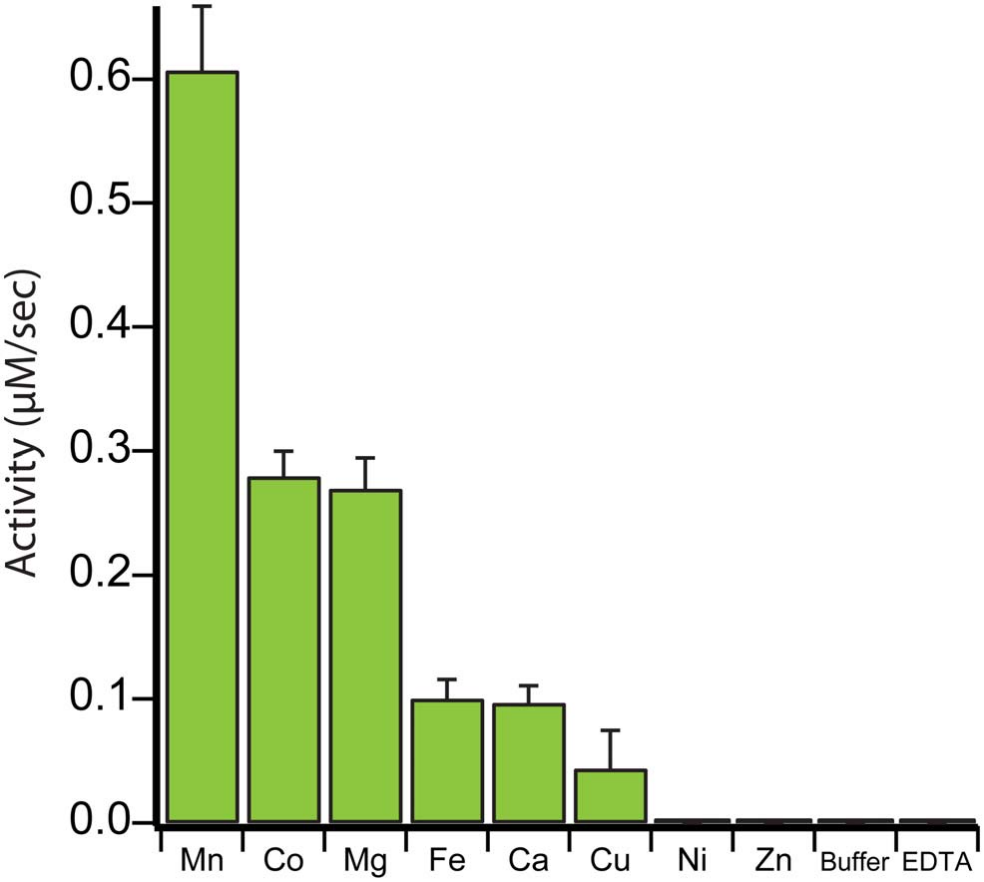
PepQ can utilize different divalent metals with varying efficiency. *E. coli* PepQ (50 nM) was assayed in the presence of various metals, in the absence of added metal (Buffer) or in the presence of EDTA. All metals used were in the form of metal-dichlorides. Error bars show the standard deviation of three independent samples.

Despite the shared pita-bread fold, prolidases, methionine aminopeptidases and proline amino-peptidases from a range of taxa, display widely varying abilities to bind and utilize different metals for catalysis. The presence of magnesium ions in both metal binding sites of *E. coli* PepQ (Figure 2) suggests that this prolidase might be enzymatically active with this metal, though magnesium is not known to be the preferred metal of any pita-bread fold enzyme. We therefore examined the ability of PepQ to utilize various divalent cations – testing the dominant ions found in pita-bread fold proteases: manganese, zinc, cobalt, iron, nickel, copper, magnesium and calcium (Figure 3). As with many proteins in this family, manganese appears to be the optimal metal for PepQ activity, with cobalt a distant second. Nickel and copper are not generally employed by this family of proteases. Other metals, such as zinc and calcium, are known to require specific coordination and spacing regimes that are not easily accessed in the active site of many pita-bread fold proteins [34], leading to little or no activity, consistent with our observations with PepQ (Figure 3). Magnesium, a metal that only rarely conveys activity in other pita-bread proteases, displayed significant levels of activity with PepQ, similar to cobalt. As expected for a metalloprotease, the addition of EDTA abolished the activity of PepQ.

To control for contaminating metal in the buffer, as well as for metal that was not removed from the active site prior to the experiment, PepQ was also tested in buffer in the absence of any residual metal (Figure 3). An absence of enzymatic activity indicates that the pre-incubation of PepQ with EDTA effectively stripped any remaining bound metal. Whether the metals that convey little or no activity do not bind, or bind, but are incapable of supporting catalysis, is unknown. Zinc, for example, has been shown to bind to the active site of some pita-bread fold peptidases and still not convey activity [2], [5], [31], [34]. It is possible that the inactivity of PepQ in the presence of zinc and nickel is the result of weak metal binding, which could, in principle, be examined by increasing in the concentrations of these metals in the PepQ assay. However, the concentrations of these metals are not thought to be higher *in vivo* than used here *in vitro* [42], implying that these metals are not likely used to support catalysis in the cell.

### Ionic Interactions Favor the Substrate Peptide C-Terminus

**Figure 4:**
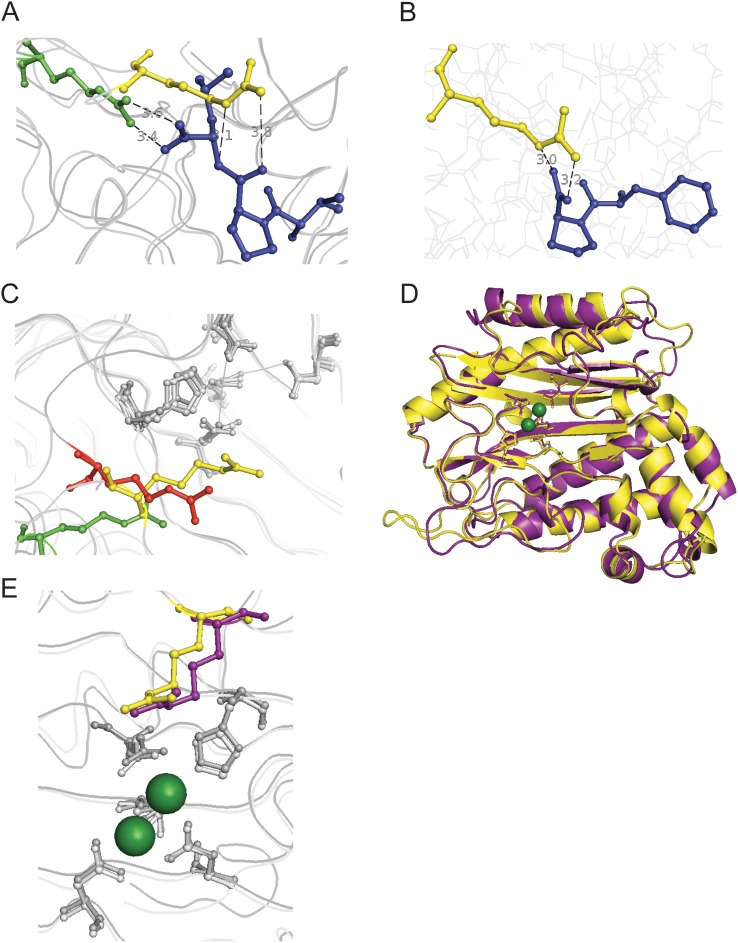
Structural alignment of prolidases reveals a conserved active site arginine. (A) The PepQ catalytic domain (residues 160–443) was aligned with *E. coli* PepP, the proline aminopeptidase, with the bound substrate tripeptide ValProLeu (PDB entry 2BHA, residues 175–425; RMSD = 1.05 Å, 1020 atoms aligned). PepQ R370 is shown in yellow, PepP R371 is shown in green and the tripeptide is colored blue. The distances between PepP R371 and the C-terminal oxygens of the tripeptide measured at 3.4 and 3.6 Å. The distances between PepQ R370 and the prolyl-leucyl amide nitrogen and oxygen measured at 3.1 and 3.8 Å, respectively. (B) Docking simulations were performed between PepQ (yellow) and substrate dipeptides using AutoDock Vina [38]. Shown is the substrate PhePro (blue). The distances between R370 and the dipeptide C-terminal oxygens measured at 3.0 and 3.2 Å. (C) *E. coli* PepQ (yellow) and PepP (green) were aligned with *P. furiosus* prolidase (PDB entry 1PV9, residues 124–345, red). PepQ R370, PepP R371 and *P. furiosus* R295 are highlighted. (RMSD*_Ecoli_*_Q–*Pfuriosus*Q_ = 0.92 Å, 816 atoms aligned; RMSD*_Ecoli_*_P–*Pfuriosus*Q_ = 0.82 Å, 908 atoms aligned) (D) Structure alignment of catalytic domains of *E. coli* PepQ (yellow) and human PepD (PDB entry 2IW2, residues 187–470, purple*;* RMSD = 0.97 Å, 1179 atoms aligned). (E) R370 in PepQ (yellow) is sequentially and structurally conserved in humans (R398, purple). All structural alignments and distance measurements were performed with PyMOL [63].

While the metal-chelating residues of prolidase are well-described, the identity of these residues does not reliably predict metal usage. Likewise, *de novo* prediction of substrate specificity is limited to dipeptides, as well as certain small molecules such as organophosphates that are hydrolyzed with far lower efficiency than peptide substrates. Different prolidases have varying affinities for dipeptides, though cleavage of collagen-catabolism products, such as GlyPro or AlaPro, seems to be conserved [3], [6], [31], [43]. How dipeptide specificity is enforced by prolidase, as well as why these enzymes display a total lack of activity toward longer peptides, is not obvious, particularly given the high structural similarity between prolidase, which cannot cleave peptides longer than two amino acids, and proline aminopeptidase, which can cleave tripeptides (Xaa-Pro-Xaa) at the N-terminal side of proline. To further examine these differences, the structures of PepQ and PepP, the *E. coli* proline aminopeptidase, were aligned for comparison (Figure 4A). In the structure of PepP, which includes a bound tripeptide, Arg371 (of PepP) interacts with the C-terminus of the tripeptide. PepQ Arg370, which is projected further into the active site on one of the loop regions conserved in prolidases from higher organisms, is placed far enough into the active site that it would physically impede the binding of longer peptides, as seen in the overlap between this arginine and the PepP-bound substrate tripeptide. PepQ R370 is, however, in an appropriate position for the guanidinium group of the arginine to interact with the C-terminus of the proline residue.

**Figure 5:**
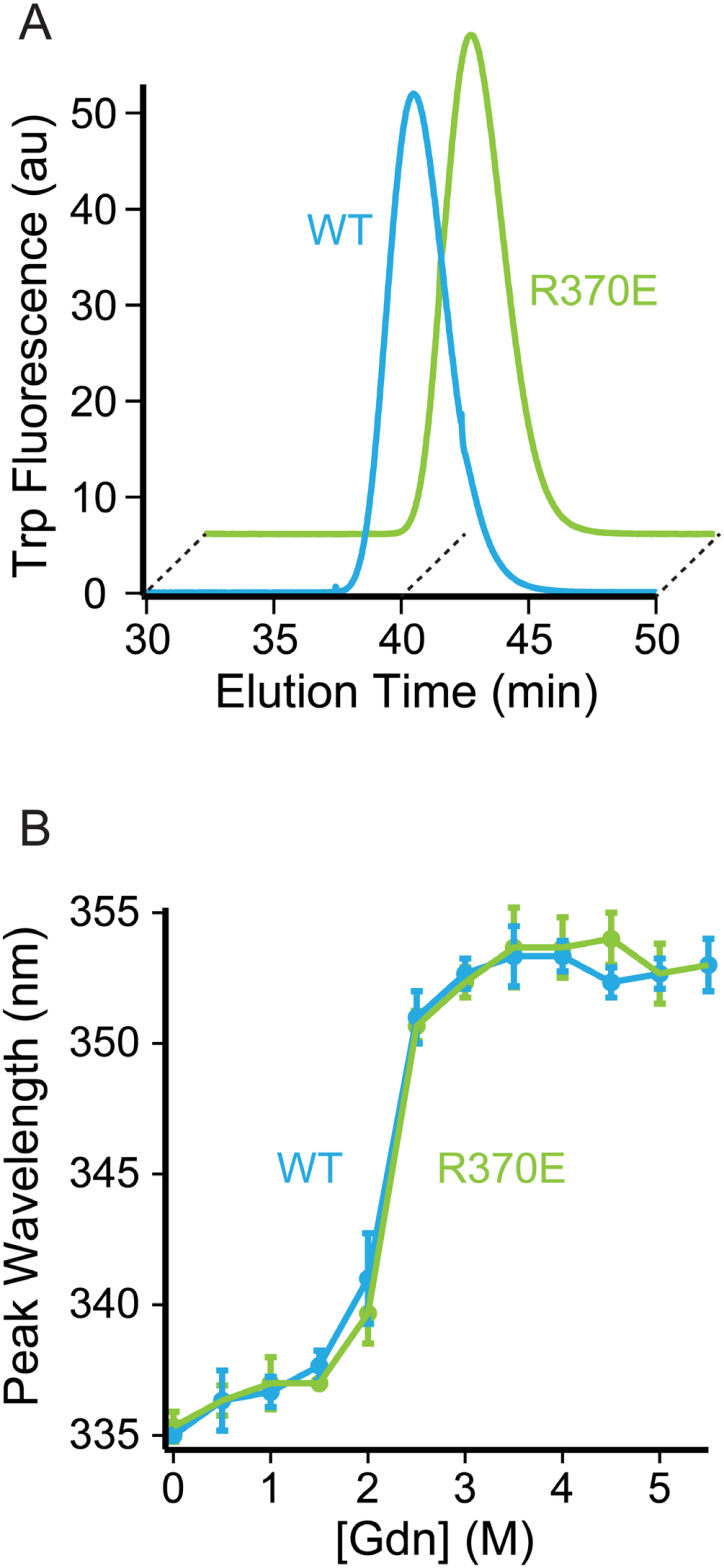
R370E mutation does not perturb PepQ structure or stability. (A) Wild-type (WT, blue) and R370E (green) PepQ (10 nM) were analyzed by analytical size exclusion chromatography. (B) Wild-type (WT, blue) and R370E (green) PepQ (50 nM) were incubated with varying concentrations of guanidiunium-HCl and the peak position of the tryptophan fluorescence emission spectrum of each was determined. Error bars indicate the standard deviation from three independent samples.

To further examine the potential role of R370 in dipeptide selection by PepQ, the structures of model dipeptides were docked into the active site of the PepQ structure [38]. Docking of dipeptides resulted in a configuration similar to that seen in PepP – the terminus of the dipeptide is in position to interact with Arg370 (Figure 4B). In order to experimentally test this interaction, a two-pronged approach was pursued. First, a charge-reversed mutant of PepQ (R370E) was made in which the predicted favorable interaction between the peptide carboxylate and R370 was replaced with an unfavorable interaction. The R370E mutant was expressed and purified following the protocols used for the wild-type enzyme, and eluted on gel filtration chromatography identically to wild-type PepQ (Figure 5A), suggesting that both the structure and dimer stability of the enzyme was not significantly compromised by the R370E mutation. To test this conclusion further, we examined the thermodynamic stability of the R370E mutant relative to wild type PepQ using guanidinium-induced unfolding at 25°C. As shown in Figure 5B, the two proteins show essentially identical unfolding transitions, indicating that the thermodynamic stability of PepQ is not affected by the R370E mutation. Consequently, any changes in the activity of R370E relative to the wild-type enzyme are not likely due to secondary effects of the mutation on protein structure or stability.

As a second approach to examining the role of R370, we examined the activity of PepQ toward a substrate dipeptide featuring a terminal amide, rather than a carboxylic acid. With this modified substrate, the predicted interaction with Arg370 should remain favorable, as hydrogen bonding could still occur, though the favorable ionic interaction would be lost. Due to the partial positive charge of the amide nitrogen, a potentially favorable ionic interaction between the modified amide terminus of this substrate dipeptide and the glutamate of the mutant R370E remained a possibility. Kinetic analysis of both wild-type and R370E PepQ with both AlaPro-COOH and AlaPro-CONH_2_ strongly supports the proposed model for the role of R370 (Table 2). R370E displayed a considerably higher K_m_ for the substrate AlaPro-COOH than the wild-type protein, while actually having a lower K_m_ for AlaPro-CONH_2_, when compared to wild-type prolidase. The reduction in k_cat_ seen in R370E is likely due the role of this residue in the organization of water and other residues in the active site [20]. The changes in K_m_, with information from both a charge-reversed protein and a charge-neutralized substrate, strongly suggest an interaction between the substrate carboxylate group and R370.

**Table 2:** Kinetic parameters for the hydrolysis of the dipeptides AlaPro and AlaPro-NH_2_ by wild-type and R370E PepQ.

	AlaPro-COOH			AlaPro-CONH_2_		
	k_cat_/Km(M^−1^s^−1^)	k_cat_ (s^−1^)	K_m_ (mM)	k_cat_/Km(M^−1^s^−1^)	k_cat_ (s^−1^)	K_m_ (mM)
**WT**	1.2×10^5^	139.1±2.5	1.2±0.1	1.2×10^4^	80.7±19.7	6.6±0.5
**R370E**	5.1×10^1^	6.5±1.1	127±2	2.3×10^2^	1.1±0.4	4.7±0.2

### The placement of the loop arginine evolved for substrate selectivity

The location of the key R370 residue in PepQ, and similar arginine residues in other pita-bread fold enzymes, may have been an important factor in the evolution of prolidase. To examine this idea, the structures of *E. coli* PepP, *E. coli* PepQ and *P. furiosus* PepQ were aligned (Figure 4C). The loop region containing this arginine is absent from *P. furiosus* prolidase. While the archaeal prolidase retains an arginine in the same spatial location of the active site (Arg295), it appears to be in an intermediate position, relative to PepP and PepQ from *E. coli*. The active site residues of the three enzymes are nearly super-imposable, indicating that this change in position is not an artifact of the structure alignment. It thus seems reasonable that the addition of the expanded peptide regions containing the arginine in Gram-negative bacteria and eukaryotes could have resulted from evolutionary fine tuning of the enzyme for high specificity dipeptidase activity. While the loop residues are conserved in the sequence of human prolidase, we sought to verify the placement of this residue as a potential means of selecting for dipeptides. The structures of the catalytic domains of human and *E. coli* prolidases were aligned, showing high conservation in the secondary structure elements and an RMSD of less than 1Å (Figure 4D, secondary structures shown in Figure 1). The critical arginine is observed in the active site of the human prolidase in a position nearly identical to the bacterial residue (Figure 4E). This suggests that after the initial evolution of the loop regions for the placement of this residue, no further optimization was necessary for the selection of dipeptides as the enzyme evolved further over the course of several billion years.

## Discussion

The results presented here support a role for substrate length specificity in pita-bread fold enzymes through the positioning of an active site arginine. With a high-resolution structure of *E. coli* PepQ in hand, we were able to compare it to related enzymes, both bioinformatically and structurally. We found that the position of the active site arginine has changed during the evolution in this family of proteins, with a shift further into the active site leading to selection against peptide substrates greater than two residues in length. Not only does the placement of this arginine physically occlude longer peptides, as seen structurally, but also, kinetic analysis demonstrates the important role of the ionic interaction between this positively charged residue and the negatively charged C-terminus of the substrate dipeptide. We have also found that while this protein is maximally active with manganese, it can utilize other metals, including magnesium, an uncommon property for this family of metalloproteins.

Although they are very similar proteins, the members of this family of enzymes vary in a number of significant ways. Of particular note is the presence of several large regions of additional residues in the prolidase sequences of Gram-negative bacteria, single-celled eukaryotes and higher plants and animals that are absent in the sequences of other bacterial prolidases, as well as proline aminopeptidase. When comparing the additional regions found in *E. coli* that are absent in *P. furiosus*, perhaps the most striking insert is the N-terminal loop. This loop not only makes significant contact with its counterpart on the adjacent subunit, but also contributes to the opening of the active site, relative to the loop-less *P. furiosus* structure. The role of the two helices inserted into the catalytic domain of *E. coli* is more difficult to surmise, given the distance from the active site and the other subunit. The structural rearrangements created through the insertion of the N-terminal loop may be stabilized by the presence of these helices, but this remains to be examined. However, both of these helices, as well as the N-terminal loop are found in the sequence and structure of *E. coli* PepP (Figure 1) [22], [30], [40]. This suggests that these changes may have occurred in an ancestor of this family before the divergence that led to separate substrate specificities of PepQ and PepP. The component found in neither PepP nor *P. furiosus* PepQ is the loop in the active site of the protein, which contains the conserved arginine.

Enzymes generally dictate specificity by utilizing binding pockets with specific interactions that favor some substrates and disfavor or occlude other substrates [44]–[47]. Pita-bread fold proteins are no exception – the occlusion of branched amino acids and selection against small amino acids in substrates has been observed previously in PepP [34] and charge interactions have been observed to dictate specificity in some prolidases [48]. Despite the high level of conservation among proteins with the pita-bread fold, these enzymes are very specific for their substrates, at least in terms of peptide length. While the evolutionary benefit of selecting for dipeptides stems from the availability of byproducts of protein catabolism, like those derived from collagen, the movement of this residue also levies an advantage against certain small molecules. *E. coli* PepQ can not only hydrolyze at least thirteen different dipeptides, but also an assortment of organophosphates and other small molecules [6]. While these substrates vary considerably on the N-terminal side of the scissile bond, the C-terminal end of all previously tested substrates shared a negatively charged group, either a carboxylate or a nitro group [6]. Reactivity toward these substrates is likely dictated by the positioning of R370 in the active site of the enzyme. We have shown that the addition of a loop in the catalytic domain, near the active site, allowed for the substrate peptide length-determining residue to be repositioned, altering the specificity of the enzyme. Utilization of an arginine at the designated position in either PepP or PepQ for this selection likely stems from the ability of arginine to interact ionically with both oxygens in the C-terminus of the substrate peptide, as well as through hydrogen bonding. A lysine at this position is unlikely to interact with both oxygens due to spatial and angular limitations. Although the enzyme is still functional without this interaction, the activity is severely compromised, which is consistent with reports that *E. coli* PepP has minor activity against dipeptides [18]. Interestingly, the genomes of sequenced *Pyrococcus* species include PepQ sequences, but lack PepP annotations [49]–[55]. Given the intermediate positioning of the conserved arginine in *P. furiosus* PepQ, a dual functionality for cleaving di- and tripeptides may be predicted for that enzyme.

While many prolidases share various similarities, structural and biochemical data reveal that *E. coli* prolidase is more similar to the human enzyme than other enzymes. The catalytic domains of the *E. coli* and human prolidases align with an RMSD of less than 1.0 Å, and this bacterial enzyme utilizes magnesium to a similar extent as human prolidase, suggesting that the specific placement or conformational flexibility that influences metal coordination is shared between the enzymes of these two distantly related organisms. Variable metal usage has been postulated to serve a regulatory role in aminopeptidases [29]. Other similarities between these two proteins may allow for the *E. coli* protein to provide insights into the functionality of the human protein. Not only is the placement of the critical arginine residue unchanged in the human prolidase, but many other residues are conserved between the two proteins, including some that are associated with disease alleles, for example, E412K and G448R in human prolidase deficiency [41].

Interestingly, despite its role in substrate selectivity, no mutation of the equivalent R370 residue has yet been associated with the onset of prolidase deficiency in humans [13], [56]–[58]. It is possible that mutation of the same residue in the human enzyme results in a reduction of enzymatic activity too small to yield an observable phenotype. However, this seems unlikely, given that losing R370 in PepQ results in a decrease in enzymatic activity that is orders of magnitude more severe than caused by single residue mutants in the human enzyme with known phenotypes [41]. Notably, many disease associated mutations also (i) decrease the stability of the enzyme, (ii) have a reduced abundance *in vivo*, and (iii) perturb the dimer binding constant so that formation of active enzyme requires protein concentrations that are much higher than needed for the wild-type protein [41]. It is possible that the impact of these mutations on folding and stability is, overall, more serious than the loss of activity seen with the arginine mutation alone, which has no effect on enzyme stability or folding. Alternately, loss of the active site arginine might have such severe developmental consequences that homozygous and many heterozygous genotypes are simply not viable. It also remains possible that the number of studied cases of prolidase deficiency is yet too small to have sampled every disease-associated allele. Although the critical active site arginine residue has yet to be associated with the physiological outcomes of reduced activity, observed defects in conserved regions in one enzyme generally predict similar defects in other, highly homologous enzymes. Utilizing *E. coli* PepQ may, therefore, be an effective strategy for studying prolidases in general and deficiencies in human prolidase specifically.

Many proteins evolve through the addition of loops or domains to gain solubility, new interactions or new activity [59]. Although it is not necessary for a prolidase to have the additional catalytic domain loop in order to place specificity-defining residues in the active site, as seen in the *P. furiosus* PepQ, the positioning of this residue in Gram-negative bacteria and higher organisms has remained constant during billions of years of evolution, indicating a preferred or optimal placement for activity. While *E. coli* PepQ may serve as a tool for studying prolidases in general, other prolidases may provide further insight into the role of these enzymes beyond collagen recycling in humans. One such protein of interest is the Xaa-Pro peptidase from the nearly exclusively catabolic organism *Mycoplasma mobile*. Examination of this protein structurally and biochemically would reveal how this minimalist organism utilizes this enzyme, demonstrating the extent of its role in metabolism. Despite the continual advance of knowledge about prolidases – structurally, biochemically and genetically – much is left unknown about the role of these enzymes in metabolism and their connection to disease.
